# *In Vitro* Digestion and Fermentation of Cowpea Pod Extracts and Proteins Loaded in Ca(II)-Alginate Hydrogels

**DOI:** 10.3390/foods13193071

**Published:** 2024-09-26

**Authors:** Maria Victoria Traffano-Schiffo, Tatiana Rocio Aguirre-Calvo, Beatriz Navajas-Porras, María Victoria Avanza, José Ángel Rufián-Henares, Patricio Román Santagapita

**Affiliations:** 1 Instituto de Química Básica y Aplicada del Nordeste Argentino, IQUIBA-NEA, UNNE-CONICET, Avenida Libertad 5460, Corrientes 3400, Argentina; m.traffanoschiffo@conicet.gov.ar (M.V.T.-S.); maria.victoria.avanza@comunidad.unne.edu.ar (M.V.A.); 2 Departamento de Química Orgánica y Química Inorgánica, Facultad de Ciencias Exactas y Naturales, Universidad de Buenos Aires, Buenos Aires 1428, Argentina; traguic@gmail.com; 3 Centro de Investigaciones en Hidratos de Carbono (CIHIDECAR), Universidad de Buenos Aires-CONICET, Buenos Aires 1428, Argentina; 4 Departamento de Nutrición y Bromatología, Instituto de Nutrición y Tecnología de Alimentos, Centro de Investigación Biomédica, Universidad de Granada, 18011 Granada, Spain; beatriznavajas@ugr.es; 5 Instituto de Investigación Biosanitaria ibs.GRANADA, Universidad de Granada, 18014 Granada, Spain

**Keywords:** *Vigna unguiculata*, bioactive compounds, antioxidant capacity, gut microbiota, low-field nuclear magnetic resonance, short-chain fatty acids

## Abstract

Antioxidants derived from food by-products are known for their bioactive properties and impact on human health. However, the gastrointestinal behavior is often poor due to their degradation during digestion. The development of Ca(II)–alginate beads supplemented with biopolymers and enriched with cowpea (*Vigna unguiculata*) extract could represent a novel environmentally friendly technological solution to produce functional ingredients in the food industry. The present study evaluates the impact of *in vitro* digestion/fermentation by analyzing global antioxidant response (GAR), production of short-chain fatty acids (SCFAs) as a modulation of gut microbiota, and behavior of proton transverse relaxation times by low-field nuclear magnetic resonance (as an indicator of gelation state and characterization of microstructure). Results revealed that guar gum and cowpea protein preserved a high GAR of total phenolic compounds and antioxidant capacity by ABTS and FRAP methods after digestion/fermentation, promoting an adequate protection of the bioactives for their absorption. Alginate-based beads have great potential as prebiotics, with the guar gum-containing system contributing the most to SCFAs production. Finally, the overall higher mobility of protons observed in the intestinal phase agrees with structural changes that promote the release of phenolic compounds during this stage. Beads are excellent carriers of bioactive compounds (cowpea phenolic compounds and peptides) with potential capacities.

## 1. Introduction

Free radicals in the human body are produced as by-products of metabolic pathways due to the oxidative stress triggered by internal and/or external factors, such as pollution, radiation, ozone, medication, cigarette smoking, and stress [1]. These highly reactive and unstable compounds can cause significant cell damage and homeostatic disruption, leading to serious diseases, including cancer, and heart, intestine, liver, prostate, lung, and neurodegenerative diseases [2,3]. Antioxidant compounds are stable molecules that can interact with free radicals in a safe manner, terminating the chain reaction and converting them to harmless molecules, thereby protecting the human body against its action [4]. Antioxidants are commonly produced endogenously by body metabolism through enzymatic or non-enzymatic pathways for defense and protection. However, in most cases, production is insufficient to completely prevent oxidative damage [3,4,5]. This insufficiency is compensated by the incorporation of exogenous antioxidants through diet or food supplements [6] that are mainly recommended to reduce oxidative damage to the human body.

Cowpea (*Vigna unguiculata*), a legume crop known for its nutritional and medicinal properties, contains phenolic compounds (anthocyanins, flavonols, and free phenolic acids, among others) [7], high protein content (24–27% (*w*/*w*), including globulins, albumins, prolamins, and glutelins), essential amino acids (Thr, Val, Ile, Leu, Lys, His, and Arg, among others) [8], and starch [9]. Research related to the extraction of phenolic compounds, their uses, and health-promoting properties [10] from different parts of cowpea crops, such as leaves [11,12], seeds, and pods [13,14], has been conducted in recent years. Phenolic compounds from cowpea pod extracts obtained by the application of green extraction technologies (such pressurized liquid extraction) [15] were identified—for example, gallic acid, caftaric acid, *p*-coumaric acid, quercetin, kaempferol, ferulic acid, several compounds derived from hydroxybenzoic acid, and high contents of hydroxylated and methoxylated benzoic and cinnamic acids, along with tetrahydroxylated flavones and flavonols. Nevertheless, the benefits of the incorporation of these compounds in the diet will be possible only if they withstand several steps, such as food processing, controlled release from the food matrix after ingestion, bioavailability in the gastrointestinal tract, metabolic changes, and disposition/absorption of the molecules in the tissues of interest [16].

An effective route for overcoming these complexities is encapsulation into biopolymers, which preserves bioactive ingredient characteristics, leading to an improvement in stability and bioaccessibility [17] while offering prolonged and controlled release of bioactive compounds [18]. Among biopolymeric agents, alginate hydrogels have been reported to enhance stability against processing and gastric conditions (pH and proteolytic enzymes) for a number of water-insoluble and micro-particulate encapsulated compounds [19,20]. Alginates have been widely used as an immobilization matrix for encapsulation with controlled delivery applications. The synthesis of Ca(II)–alginate beads is based on ionotropic gelation and is easy to carry out, inexpensive, and offers mild conditions to bioactives [19]. Often, other excipients, such as gums (arabic, guar), pectins (low ang high methoxyl), and sugars (trehalose and sucrose), are included since they improve the protection of bioactives and modulate the nano- and micro-structure [21,22,23]. In a previous work, we optimized the extraction of phenolic compounds with antioxidant capacity from cowpea pods and encapsulated them in Ca(II)–alginate hydrogels, focusing on the microstructural changes (alginate network, alginate rods, and dimers from alginate) produced by the addition of hydrocolloids (like guar and arabic gum) or isolated cowpea proteins [13]. The properties that biopolymers provided to Ca(II)–alginate beads (such as mechanical strength or release behavior) were strongly defined by structural characteristics [21,22,23].

The aim of this research was to encapsulate cowpea pod extract in alginate systems with different formulations (containing biopolymers and isolated cowpea protein) to assess the impact of *in vitro* digestion and fermentation on the formulated beads. Furthermore, antioxidant capacity and total phenolic compounds were evaluated during digestion and after fermentation, as well as the production of short-chain fatty acids (SCFAs) as a proxy for the modulation of gut microbiota. Moreover, proton mobility was monitored during digestion by LF-NMR as an indicator of the state of interactions on the Ca(II)–alginate gel.

## 2. Materials and Methods

### 2.1. Materials

To synthesize alginate systems, the following materials were used: sodium alginate: 1.97 × 10^5^ g/mol; M/G ratio: 0.6 (Cargill S.A., San Isidro, Buenos Aires, Argentina); arabic gum: 250,000 g/mol (Biopack, Zárate, Buenos Aires, Argentina); guar gum: 220,000 g/mol, mannose/galactose ratio: 1.8, protein content of 2.1 ± 0.3 g/100 g dry gum (Cordis S.A., Villa Luzuriaga, Buenos Aires, Argentina); and isolated cowpea protein from seed obtained at pH = 10 [24]. The reagents used for *in vitro* digestion and fermentation were salivary α-amylase, pepsin, extract of porcine bile acids, sodium dihydrogen phosphate, sodium sulfide, resazurin, tryptone, and cysteine obtained from Sigma-Aldrich (Darmstadt, Germany), and pancreatin (porcine pancreas) from Alpha Aesar (Kandel, Germany). Reagents and techniques used for antioxidant properties were purchased from Sigma-Aldrich (St. Louis, MO, USA) (Section 2.4.1. and Supplementary File).

### 2.2. Extract Preparation and Encapsulation

The extract was prepared using cowpea pods (Colorado varieties provided by the Instituto Nacional de Tecnología Agropecuaria (INTA), El Sombrero, Corrientes, Argentina) and applying high-intensity ultrasound, as previously optimized by Traffano-Schiffo et al. [13]. Briefly, the cowpea mixture (cowpea pod flour and distilled water in a 1:15 ratio) was sonicated for 10 min at 36% amplitude with a 13 mm probe (220-B, model CV334, Sonics, Newton, CT, USA) at a frequency of 20 kHz and a maximum power of 500 W, connected to a processor with temperature control (VCX500, Sonics). The sonicated mixture was then centrifuged at 6000 rpm (7647× *g*) for 15 min, vacuum filtered (using Buchner and filter paper), freeze-dried at −60 °C and 0.03 mbar for 32 h in a Christ Alpha 1–4 LO freeze-dryer (Martin Christ, Osterode am Harz, Germany), and stored in darkness at 4 °C until use.

The systems were prepared with reconstituted extract (78.9 mg in 10 mL of 0.1 M acetate buffer pH 5.5) and without extract (control). Five different alginate (1.5% (*w*/*v*)) and gums (0.25% *w*/*v*) solutions were prepared for encapsulation: alginate (A), alginate–arabic gum (AAG), alginate–guar gum (AGG), and alginate–isolated cowpea protein in a 2:1 ratio (alginate:protein) (AP2:1) and 1:1 ratio (AP1:1). A summary of bead formulations is shown in Table S1. All alginate solutions (with extract and control) were dropped with a 0.4 mm pipette tip in a calcium chloride solution (2.5% (*w*/*v*) in 0.1 M acetate buffer pH 5.5) at 20 ± 1 rpm using a peristaltic pump (model 7554-95 connected to a Masterflex L/S 7518-00, Cole Parmer, Vernon Hills, IL, USA). The formed Ca(II)–alginate beads were kept in a CaCl_2_ solution for 5 min, washed, and hermetically sealed at 4 °C until use.

### 2.3. Morphology Analysis

A macroscopic analysis was performed to evaluate the impact of the formulation on the macrostructure of the beads, which in turn can affect different physicochemical properties, such as water sorption as well as consumer acceptance. Area, Feret’s diameter, circularity, perimeter, and roundness of beads were obtained from digital images using ImageJ software (1.53t version, National Institutes of Health, Bethesda, MD, USA) (https://imagej.net/ij/index.html, accessed on 4 April 2024) [25].

### 2.4. In Vitro Gastrointestinal Simulation

**Figure 1:**
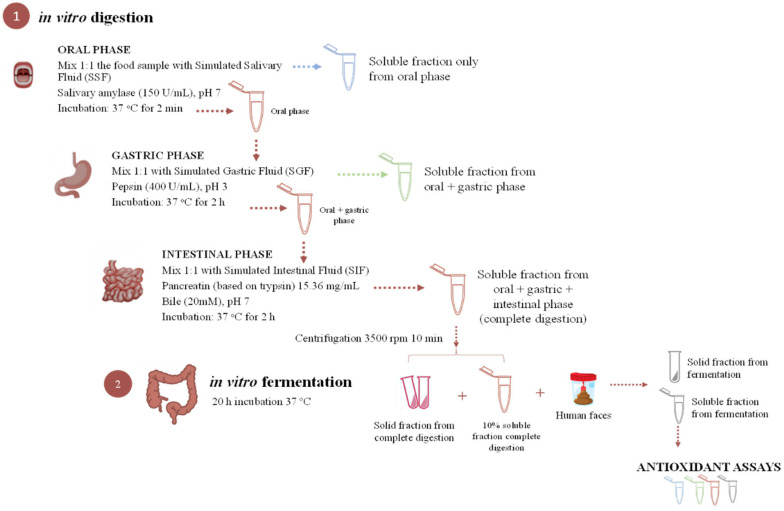
Schematic representation of the *in vitro* digestion and fermentation process.

The gastrointestinal simulation proposed by Minekus et al. [26] and Pérez-Burillo et al. [27] consists of two major protocols, as shown in Figure 1. Beads containing extract, control beads, and the non-encapsulated extract were subjected to *in vitro* digestion–fermentation. Briefly, (1) a three-step procedure, which simulates digestion in the mouth (oral digestion with simulated salivary fluid (SSF), composed of α-amylase (150 U/mL) and salts), stomach (gastric digestion with simulated gastric fluid (SGF) composed of pepsin (400 U/mL) and salts), and small intestine (intestinal digestion with simulated intestinal fluid (SIF) composed of pancreatin from porcine pancreas (15.36 mg/mL), bile salts (20 mm), and salts. In particular, for the oral phase, samples were mixed at a ratio of 1:1 (*w*/*v*) with SSF and maintained at 37 °C for 2 min in an oscillatory shaker at 30 oscillations/min. For the gastric phase, SGF was mixed in a 1:1 (*v*/*v*) ratio with the oral phase, the pH was lowered to 3 by adding 1N HCl, and then it was incubated at 37 °C for 2 h in an oscillatory shaker at 30 oscillations/min. Finally, for the intestinal phase, SIF was added to the gastric phase, and the pH was raised to 7 by adding 4 M NaOH, followed by incubation at 37 °C for 2 h in an oscillatory shaker at 30 oscillations/min. Tubes were immediately immersed in ice to stop enzymatic reactions once the intestinal phase was finished, and thereafter centrifuged at 3500 rpm for 10 min. Before each incubation, a volume of CaCl_2_ was added to each phase according to the protocol. The supernatant, which represents the fraction available for absorption in the small intestine, was stored at −80 °C until analysis. The solid fraction, which represents the non-digested fraction that enters the large intestine, was used as the *in vitro* fermentation substrate [28].

Then, (2) an *inoculum* (32% human feces in phosphate buffer) was prepared for the fermentation procedure; a final fermentation solution was mixed by combining the fermentation medium (peptone water 15 g/L) and the reductive solution (51.5 mM cysteine, 80 mM sodium sulfide, 0.04 M NaOH, and 0.01% (*w*/*v*) resazurin). The fecal material collected for the *inoculum* was provided by healthy volunteers with a normal body mass index and who did not have any pathology, nor had they taken antibiotics for at least three months prior to the donation. For the fermentation protocol, the *in vitro* fermentation substrate was weighed (0.5 g + 10% of supernatant of intestinal phase), then 7.5 mL of final fermentation solution and 2 mL of *inoculum* were added [23]. An anaerobic atmosphere was produced by bubbling nitrogen through the mixture, followed by incubation at 37 °C for 20 h in an oscillatory shaker at 30 oscillations/min. Immediately afterwards, the samples were immersed in ice to stop microbial activity and centrifuged at 3500 rpm for 10 min. The supernatant (available fraction) was collected and stored at −80 °C until analysis.

#### 2.4.1. Antioxidant Capacity Methods, Loading Efficiency, and Remaining Antioxidant Activity

Antioxidant capacity (AC) was evaluated in undigested beads, digested fractions (oral, gastric, and intestinal, separately), and fermented fraction with and without extract. To determine the global antioxidant response of *in vitro* digestion–fermentation, three different methods were used: Folin–Ciocalteu for total phenolic compounds [6,29], antioxidant capacity against ABTS*^+^ radical (2,2′-azino-bis(3-ethylbenzothiazoline-6-sulfonic acid) [30], and antioxidant capacity referring to ferric-reducing antioxidant power assay (FRAP) [31,32]. The specific protocols are detailed in the Supplementary Information.

Undigested beads were measured after liquefying 10 beads in sodium citrate (20% (*w*/*v*)) with vigorous shaking. The loading efficiency of phenolic compounds by Folin–Ciocalteu (L.E._TP_), and the remaining antioxidant activity by ABTS (R.A.A._ABTS_) and FRAP (R.A.A._FRAP_) were determined using equations from Traffano-Schiffo et al. [13]. All antioxidant assays were performed in triplicate in a 96-well microplate and measured in a microplate reader (FLUOStar Omega, BMG Labtech, Ortenberg, Germany) and monitored at 37 °C at different wavelengths (according to the method).

#### 2.4.2. Short-Chain Fatty Acids (SCFAs) Analysis

The production of SFCAs after fermentation was measured by UHPLC (ultra high-performance liquid chromatography) as described in a previous work [33]. After fermentation, the samples were centrifuged at 13,300 rpm for 5 min, the supernatants were filtered through a 0.22 μm nylon filter, and the samples were 1:10 diluted with 1 M HCl. Chromatographic separation was performed using an Agilent 1290 Infinity II LC System UHPLC-RID system (Agilent Technologies, Santa Clara, CA, USA) coupled to an Agilent Poroshell 120 SB-Aq column (Agilent Technologies, Santa Clara, CA, USA) (3 × 150 mm, 2.7 µm). The mobile phase used was 5 mM H_2_SO_4_, with an isocratic flow elution of 0.5 mL/min. The injection volume was 5 μL, and the temperature of the column and refractive index detector was 35 °C.

#### 2.4.3. Low-Field Nuclear Magnetic Resonance (LF-NMR)

The transverse relaxation time (or spin–spin) (T_2_) was measured by time-resolved low-field proton nuclear magnetic resonance in a Bruker Minispec mq20 (Bruker Biospin GmbH, Rheinstetten, Germany) with a 0.47 T magnetic field operating at a resonance frequency of 20 MHz. Beads were immersed in SSG and SIF following the protocol described in Section 2.4; after draining and slight remotion of the surface water with paper, they were placed in NMR tubes and kept at equilibrium at 37 °C in a thermal bath (Haake, model Phoenix II C35P, Thermo Electron Corporation Gmbh, Karlsruhe, Germany). T_2_ was obtained by using the Carr–Purcell–Meiboom–Gill (CPMG) sequence with the following settings: τ = 0.7 ms, scans = 16, number of points = 600, no dummy shots, and gain = 94 dB; phase cycling was used. A bi-exponential decay function was used to fit curves, as previously reported [34]. Measurements were performed in duplicate.

### 2.5. Statistical Analysis

Data are expressed as mean ± standard deviation. Two-way ANOVA with Tukey’s *post hoc* test was performed using Prism 8 (GraphPad Software Inc., San Diego, CA, USA) to determine significant differences among means for all measured parameters (*p* < 0.05).

## 3. Results and Discussion

Cowpea extract was successfully encapsulated in Ca(II)–alginate hydrogel systems by ionotropic gelation with biopolymers or isolated cowpea protein. Table 1 shows the L.E._TP_ and the remaining antioxidant activity by ABTS and FRAP of the beads before *in vitro* digestion. Beads with the higher amount of protein (AP1:1) or gums (AGG and AAG) showed significantly higher L.E._TP_ with respect to A, while the R.A.A._ABTS_ and R.A.A._FRAP_ were significantly higher for systems containing isolated cowpea protein (AP2:1 and AP1:1). In this context, the use of gums or proteins favors the encapsulation of active compounds, allowing the generation of beads as vehicles that protect the desired compounds during synthesis. The biopolymers’ capacity to stabilize the microstructure of the Ca(II)–alginate beads or to improve the mechanical stability of the systems has been extensively discussed in previous works [21,22]. On the other hand, macroscopic characteristics (Table S2) were studied for the Ca(II)–alginate beads. The area (0.05 to 0.062 cm^2^), perimeter (0.87 to 1.09 cm), and Feret’s diameter (0.27 to 0.31 cm) were similar to the results obtained for formulated Ca(II)–alginate beads [35]. Regarding circularity, beads containing isolated cowpea proteins showed the highest values, as previously observed [13].

**Figure 2:**
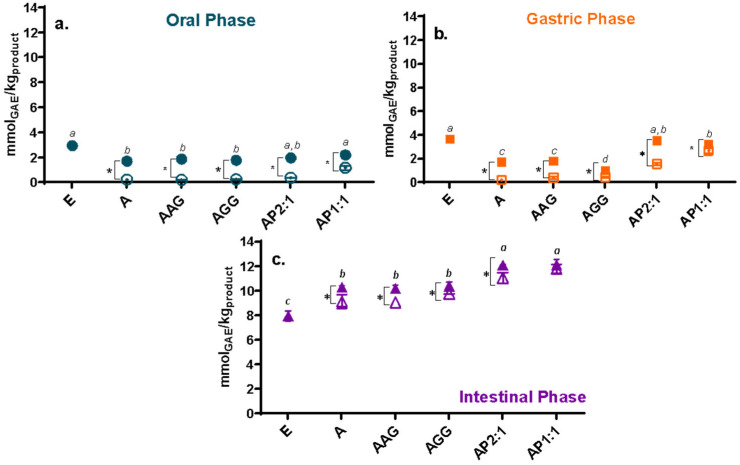
Total phenolic compounds (mmol equivalent of gallic acid per kg product) from (**a**) oral, (**b**) gastric, and (**c**) intestinal fractions of beads with extract (full symbols) and control beads (hollow symbol). Non-encapsulated extract (E) values were also included. A: alginate; AG: arabic gum; GG: guar gum; P: isolated cowpea protein in ratios of 2:1 and 1:1 (alginate:protein). Asterisk (*) indicates significant differences between samples of the same formulation with or without extract. Different lowercase letters (a–c) indicate significant differences between systems with extract (*p* < 0.05).

**Figure 3:**
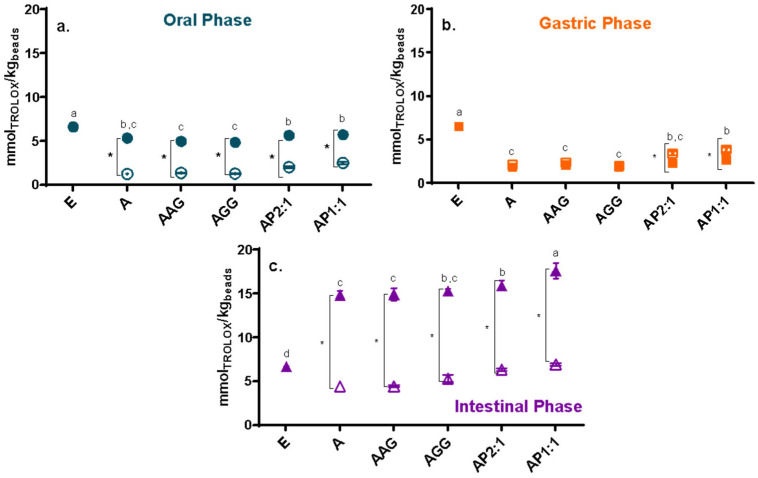
Trolox equivalent antioxidant capacity by ABTS (mmol equivalent of Trolox per kg product) from (**a**) oral, (**b**) gastric, and (**c**) intestinal fractions of beads with extract (full symbols) and control beads (hollow symbol). Non-encapsulated extract (E) values were also included. A: alginate; AG: arabic gum; GG: guar gum; P: isolated cowpea protein in ratios of 2:1 and 1:1 (alginate:protein). Asterisk (*) indicates significant differences between samples of the same formulation with or without extract. Different lowercase letters (a–d) indicate significant differences between systems (*p* < 0.05).

**Figure 4:**
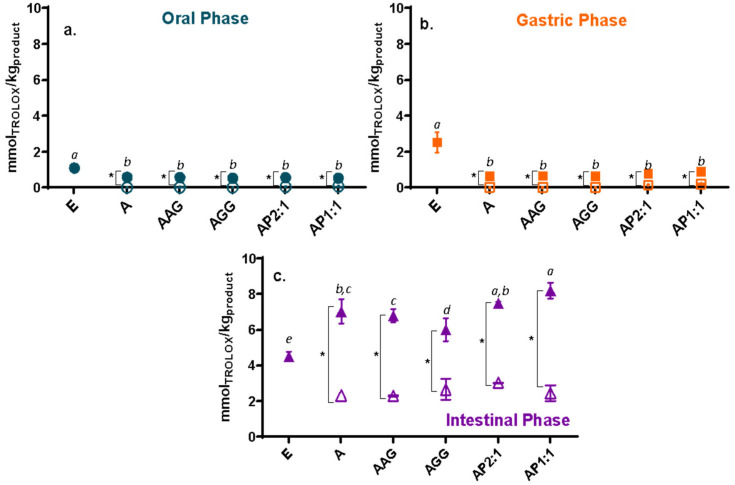
Trolox equivalent antioxidant capacity by FRAP (mmol equivalent of Trolox per kg product) from (**a**) oral, (**b**) gastric, and (**c**) intestinal fractions of beads with extract (full symbols) and control beads (hollow symbol). Non-encapsulated extract (E) values were also included. A: alginate; AG: arabic gum; GG: guar gum; P: isolated cowpea protein in ratios of 2:1 and 1:1 (alginate:protein). Asterisk (*) indicates significant differences between samples of the same formulation with or without extract. Different lowercase letters (a–e) indicate significant differences between systems (*p* < 0.05).

The total phenolic content (TPC) and antioxidant capacity (AC; determined by ABTS and FRAP) of the extract and Ca(II)–alginate beads along *in vitro* digestion are presented in Figure 2, Figure 3 and Figure 4, respectively. The phenolic content and the antioxidant capacity can change throughout the digestive tract for various reasons, such as pH, enzymes, and salt content [36]. As a general trend, the unprotected cowpea extract showed significantly higher values than the encapsulated extract in the oral and gastric phases (Figure 2a,b, Figure 3a,b and Figure 4a,b); however, the opposite trend was observed during the intestinal phase: the unprotected extract exhibited a significantly lower TPC and AC compared to the protected extract (Figure 2c, Figure 3c and Figure 4c). During physiological digestion, food disintegration mainly occurs in the mouth and stomach (oral and gastric phases), whereas enzymatic digestion and the absorption of nutrients and water occur in the intestine [37]. The TPC and AC obtained in the oral and gastric phases can be considered losses [17]. Thus, the compounds in the unprotected extract degrade, reaching the intestinal phase in significantly lower amounts than those loaded on beads.

For the oral and gastric phases (Figure 2a,b, Figure 3a,b and Figure 4a,b), loaded-extract beads and their controls showed low TPC and AC (ABTS*^+^ and FRAP) values, showing no significant release of the compounds (or their activities) in these stages (with reduced losses). Ca(II)–alginate beads are able to protect the bioactive compounds under low pH conditions (pH 3): the phenolic compounds are retained within the beads as the extra H^+^ provided by the gastric environment strengthens the structure [38], causing the beads to undergo minimal changes in this phase even at the microstructural level [23].

As a general trend, beads containing cowpea extract showed a significantly higher content and antioxidant capacities than control beads in the intestinal phase (Figure 2c, Figure 3c and Figure 4c). It should be noted that control beads showed antioxidant capacity during *in vitro* digestion, as was previously observed [23], although much less than loaded beads [39]. Regarding the studied formulations, beads containing cowpea extract showed higher activity than the unprotected extract for the three studied methods (Figure 2c, Figure 3c and Figure 4c). Thus, a significant amount of phenolic compounds and antioxidants are released in the intestine, which can be absorbed by the host to promote the desired health benefits and could be further transformed into different structures with other biological activities. Particularly, AP1:1 beads with extract showed significantly higher TPC and capacity to reduce radicals (ABTS*^+^) and ferric iron (FRAP) than the other formulations in the intestinal phase, promoted by their high solubility at intestinal pH (>80%, as shown [40]), confirming the high bioactive effect of cowpea proteins [13].

**Figure 5:**
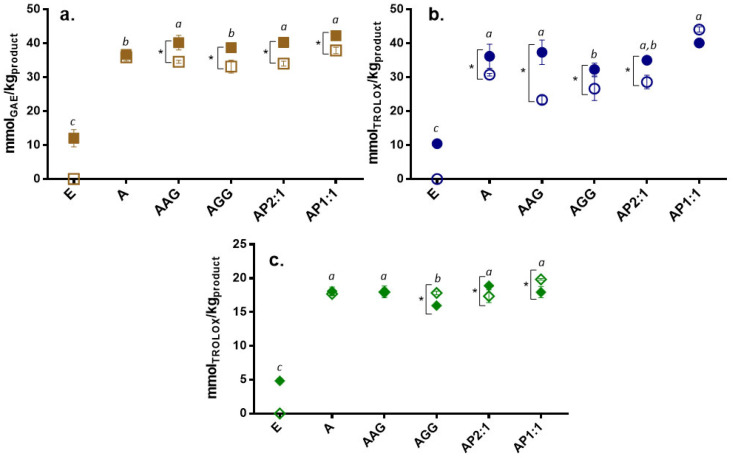
Fermented fractions of Ca(II)–alginate beads with extract (full symbols) and without extract (hollow symbols) for (**a**) total phenolic compounds and antioxidant capacity measured by (**b**) ABTS and (**c**) FRAP. Non-encapsulated extract (E) values were included. A: alginate; AG: arabic gum; GG: guar gum; P: isolated cowpea protein in ratios of 2:1 and 1:1 (alginate:protein). Asterisk (*) indicates significant differences between samples of the same formulation with or without extract. Different lowercase letters on the points (a–c) indicate significant differences between systems (*p* < 0.05).

After *in vitro* digestion, part of the intestinal phase acts as a substrate for gut microbiota. Colonic fermentation can be simulated *in vitro* by using a human fecal *inoculum* [28]. Figure 5 shows the TPC and AC (by ABTS and FRAP) of the fermented fraction of the unprotected extract and Ca(II)–alginate beads (with extract or control systems). Even though bioactive compounds in the unprotected extract reached both the intestinal and fermentative phases, their concentrations were much lower than those of any of the formulated beads, as has been previously reported [39], confirming the loss of most of its antioxidant properties in the earlier stages of the gastrointestinal tract. In contrast, all alginate formulations with encapsulated extract showed three to four times higher TPC and AC than non-encapsulated extract, highlighting the differences during fermentation with respect to the intestinal phase. The increase in antioxidant capacity during *in vitro* fermentation suggests that the microorganisms involved in this process could act on the bioconversion of phenolic compounds, favoring the bioaccessibility of these compounds [41]. This indicates that the inclusion of the extract in the beads is an accurate protection and delivery method for bioactive compounds as they reach and release the compounds at specific stages, where they will be beneficial to the host.

The Global Antioxidant Response (GAR) displayed in Table 2 involved the contribution of both the digested and fermented fractions for the loaded beads, their controls, and the unprotected extract. Beads containing the extract always had higher antioxidant capacity than the non-encapsulated extract. This is the result of the different and complex interactions that occur in the gastrointestinal environment between phenolic compounds and interact with other food constituents and enzymes, which can increase the GAR by affecting the chemical structure, molecular weight, and antioxidant solubility [42], which in turn are not only more protected in the alginate system but also are released at the time and place where it was designed. As a general trend, the antioxidant response (GAR) of control systems (without extract) was lower than that of the beads containing extract. The response was related to the components of the matrix, as extensively discussed [23]. Moreover, among Ca(II)–alginate systems, those with gums and protein as excipients had higher GAR results. Gums can optimize the encapsulation efficiency and control the release of bioactive compounds under gastrointestinal conditions, compensating for the deficiencies of beads containing only alginate [13,23]. As isolated proteins progress through the digestive process, they suffer degradation caused by enzymes and pH. These conditions induce structural changes in protein conformations and generate hydrolyzed peptides [43], leaving not only some hydrophilic groups exposed -increasing their solubility as a consequence [44], but also an increasing the number of free amino groups, which could be related to the GAR increase in beads [40]. It is worth mentioning that low-molecular-weight peptides and free amino acids from cowpea are antihypertensive [45], antidiabetic [46], anti-inflammatory, and anti-tumoral [47], representing an advantage related to the potential benefits that these beads can provide to the host.

**Figure 6:**
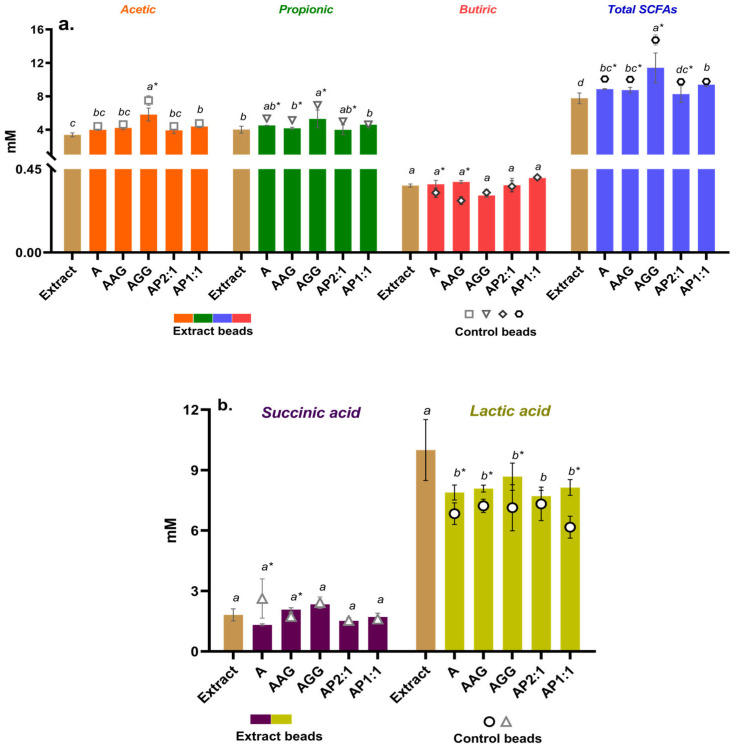
Concentration of fermentative end metabolites for Ca(II)–alginate beads with cowpea extract containing excipients. Control beads without extract and non-encapsulated extract were also included. (**a**) Short-chain fatty acids or SCFAs (mM) and (**b**) lactic and succinic acid (mM). A: alginate; AG: arabic gum; GG: guar gum; P: isolated cowpea protein in ratios of 2:1 and 1:1 (alginate:protein). The name of the acids is indicated in each column group. Different lowercase letters (a–d) indicate significant differences between Ca(II)–alginate systems with extract. Asterisk (*) indicates significant differences between control beads and extract beads (*p* < 0.05) (with and without extract). E was compared to beads with extract (*p* < 0.05).

Several metabolites are obtained at the end of the fermentation process, such as short-chain fatty acids (acetic, propionic, and butyric acids), volatile fatty acids, lactic acid, succinic acid, ethanol, methane, carbon dioxide, hydrogen, and hydrogen sulfide [48]. The production of these metabolites depends, in part, on the type of microorganisms present in the host, as well as on the type of substrate (food intake). Moreover, they are produced by specific colonic anaerobic bacteria and can confer benefits to host health [49]. SCFAs are metabolites derived from microbial fermentation of dietary fibers through their absorption and metabolism; foodstuffs that are not digested in the upper digestive tract therefore elicit energy to the host [50]. Figure 6a shows the SCFAs (acetate, propionate, and butyrate) released after the fermentation of Ca(II)–alginate beads containing cowpea extract. SCFAs production follows the same trend where acetate < propionate < butyrate as in other studies [23,39,51]. On the other hand, the unprotected extract reached colonic fermentation and produced SCFAs, but in a lower proportion than the encapsulated extract in the different systems.

Related to the specific SCFAs, acetic acid serves as an energy source for the gut peripheral cells and the liver and behaves as a signaling molecule during lipogenesis and gluconeogenesis [52]. The propionic acid pathway involves the fermentation of dietary fiber and proteins, is transported through the portal vein to the liver, and is used for gluconeogenesis and the suppression of cholesterol synthesis [53,54]. Similarly, butyric acid also serves as an energy source for colonic epithelial cells and regulates apoptotic pathways, preventing colon cancer [55] and type 2 diabetes [56]. It is important to keep in mind that the different groups of intestinal bacteria of the host exhibit different patterns of SCFAs formation related to the preferred substrates in relatively specialized metabolic niches. As reported in [57], the main fermentative microorganisms for the formation of acetate are *Bacteroides*, *Bifidobacteria*, *Lactobacilli*, *Enterobacteria*, etc.; for propionate, *Bacteroides*, *Propionibacteria*, *Clostridia*, etc.; and for butyrate, *Roseburia*, *Faecalibacteria*, *Clostridia*, *Fusobacteria*, etc. These microorganisms play a key physiological role connecting intestinal microbiota and the host [58]. According to the obtained results (Figure 6a), alginate systems containing guar gum significantly increased the production of acetic and propionic acids compared to the other systems. However, the release of butyric acid showed no significant differences between the formulations. Both carbohydrate and protein fermentation can contribute to the increase in SCFAs, but carbohydrates tend to produce higher amounts of these acids in the colon [57]. Guar gum generates large quantities of SFCAs [59], mainly propionic (~3 mM) and butyric (~4 mM) acids in gut microbiota of male and old male donors, which are closely related to the development of the intestinal immune system and its barrier function, but the specific production will depend on the type of microbiota and the interactions between food components, enzymes, and so on, as previously discussed.

Figure 6b shows the production of lactic and succinic acids after *in vitro* fermentation of beads with cowpea extract and control beads. Lactic and succinic acids are products of prebiotic fermentation and are involved in the cross-feeding—the sharing of metabolites between different microbes—of intermediary metabolites by gut bacteria [60,61], which has an emergent role in establishing robust communities. They also play a role as SCFAs precursors [62]. The encapsulated systems showed no significant differences among themselves; however, significant differences were observed between the control and beads containing extract, which may be related to the high production of lactic acid produced in the presence of the extract (as shown for the non-encapsulated extract). Concentrations of lactic acid of around 8 mM agree with the results in [63]; lactic acid exerts a positive effect in the colon as substrates and/or precursors in the production and further metabolism of SCFAs. As for succinic acid, even though there was no significant difference in the systems studied, its production has been the focus of attention due to its relevant role as a pro-inflammatory mediator in intestinal inflammation and as a profibrotic marker [62].

**Figure 7:**
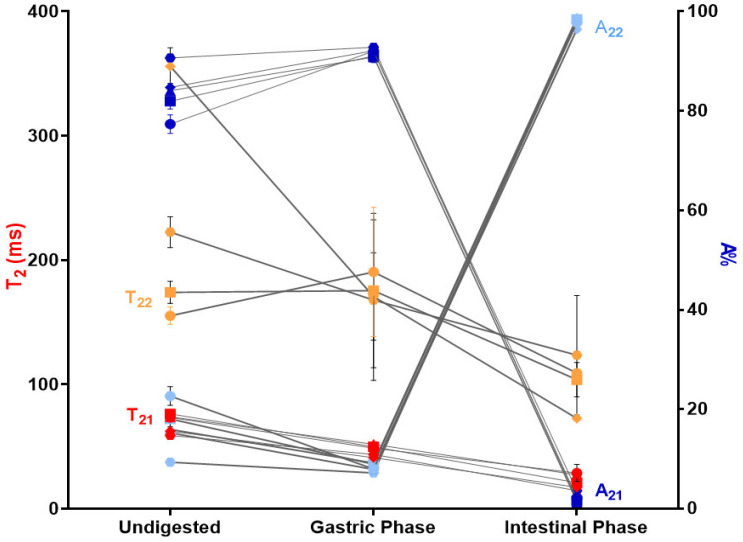
Evolution of transversal relaxation times (T_21_ and T_22_—red and orange symbols, respectively) and amplitudes (A_21_ and A_22_—blue and light blue symbols, respectively) for undigested, gastric, and intestinal samples of Ca(II)–alginate beads containing cowpea extract; (●) represents EA, (■) EAAG, (▲) EAGG, (◆) EAP2:1, and (⬣) EAP1:1. The statistical analysis is included in Table S3 (Supplementary Materials).

Transverse relaxation times (T_2_) for Ca(II)–alginate systems were monitored throughout digestion since they are a sensitive indicator of the state of gelation and a powerful tool for characterizing the microstructural properties of gels [20]. The T_2_ behaviors of the undigested, gastric, and intestinal phases of the Ca(II)–alginate beads are shown in Figure 7 and Table S3 of the Supplemental Information. In general, two proton populations (T_21_ and T_22_) with low and high mobility are observed in Ca(II)–alginate hydrogels [34,64]. A_21_ and A_22_ correspond to the intensity of the signal and represent the percentages of protons in each of these two populations (T_21_; T_22_). Those protons are mainly from water in a gel phase interacting with the solid constituents of the matrix (alginate, gums, or protein); moreover, it is important to keep in mind the complex microstructure of the Ca(II)–alginate network formed by the interconnected rods (with characteristic diameter and density), and that each digestion phase involves changes in the molarity of the media, which in turn will affect mobility.

It was expected that the extra protons from the gastric phase would produce reinforcement of the gel network at a microstructural scale of the Ca(II)–alginate beads, as demonstrated by SAXS measurements [23]. In this sense, a reduction in both T_2_ (from 59–98 to 36–55 ms, and from 155–356 to 171–288 ms in UN and GP, respectively) was also expected due to the concentration of the ions/biopolymers produced by the shrinkage of Ca(II)–alginate hydrogels in the acidic condition [20], as well as by interchange with the GP solution of higher molarity (from 32.93 to 96.37 mmol/L). On the other hand, a concomitant increase in the population of lower T_2_ was observed for all formulations (from 75–91 to 89–93% for UN and GP, respectively), in line with the shrinkage and release of water from the larger pores containing less tightened water. However, the scenario radically changed in the intestinal phase; almost all protons had the same mobility (T_22_ accounting for 96–99% of protons), showing nearly no differences along the gel network. The overall T_2_ value was less than that obtained in previous fractions (155–356 ms < 171–288 ms < 73–141 ms for UN, GP, and IP, respectively) as the molarity of the SIF solution increased (from 96.37 to 140.33 mmol/L). Moreover, because SIF contains phosphates and carbonates, Ca^2+^ ions were extracted from the network, producing a partial loss of the microstructure (as shown by SAXS determinations [23]); in particular, the Ca(II)–alginate rods were reduced both in size and in density. It seems that this rearrangement also modified the internal structure, making it more homogeneous from a mobility perspective (from two proton populations to one), generating a more interconnected network (as supported by the increase in the interconnection of the rods—from α_1_ of the SAXS measurements [23]). Then, it was expected that the reduction in T_2_ and the increase in rods interconnection would lead to an increase in the strength of the beads under SIF, as shown in Figure S1 in the Supplemental Information. Moreover, the well-known swelling of beads from acidic to neutral or slightly basic media is also in agreement with this behavior.

Even though LF-NMR analysis provides an overall picture of the mobility without individualizing each component, it is interesting to analyze the relationship between changes in mobility and the release of phenolic compounds with antioxidant activity during digestion. The overall higher mobility scenario observed in SIF is consistent with the release of phenolic compounds with antioxidant activity during this stage (Figure 2c, Figure 3c and Figure 4c). Moreover, a population of protons with reduced T_2_, accounting for the majority of protons in SGP, correlated with the low release observed in Figure 2b, Figure 3b and Figure 4b.

## 4. Conclusions

The findings obtained in the present work suggest that the formulated beads act as a protective barrier of bioactive compounds with antioxidant properties from the early stages of digestion until the release in the final stage (intestinal phase and colonic environment). Among the studied systems, there was a tendency for the gut microbiota to generate SCFAs, revealing a promising insight into the formulated beads. The potential of alginate-based beads as prebiotics has been demonstrated, with the guar gum-containing system being the one that generated the greatest contribution to the total production of SCFAs. Moreover, beads containing cowpea proteins could be a valuable alternative considering the GAR and the specific activities of the low-molecular-weight peptides and free amino acids from cowpea, representing an advantage related to the potential benefits that these beads can provide to the host. Despite these promising results, more studies (focused on different populations with different gut microbiota) are necessary.

Furthermore, the changes in molecular mobility obtained from LF-NMR data agree with the results obtained throughout digestion, demonstrating the feasibility of this encapsulation system from both structural and functional points of view. Finally, encapsulation of cowpea extracts offers promising opportunities for the integration of valuable compounds obtained from by-products in the food industry. Its encapsulation in Ca(II)–alginate beads as a delivery system could contribute to the sustainable development of new functional ingredients or even functional food (with potential anti-tumoral, anti-hypertensive, and antioxidant properties), which will be addressed in future studies.

## Figures and Tables

**Table 1:** Loading efficiency of total phenolic compounds (L.E._TP_) and remaining antioxidant activity (R.A.A) of ABTS and FRAP of beads containing cowpea pod extract.

System	L.E._TP_ (%)	R.A.A._ABTS_ (%)	R.A.A._FRAP_ (%)
A	19 ± 1 ^c^	36.4 ± 0.6 ^c^	1.9 ± 0.5 ^d^
AAG	49 ± 2 ^a^	32 ± 1 ^d^	7 ± 1 ^c^
AGG	45 ± 3 ^a^	25 ± 3 ^e^	4.5 ± 0.6 ^c,d^
AP2:1	36 ± 1 ^b^	55 ± 2 ^a^	18 ± 1 ^b^
AP1:1	47 ± 3 ^a^	47 ± 3 ^b^	26 ± 3 ^a^

A: alginate; AG: arabic gum; GG: guar gum, and P: cowpea protein. Standard deviation values are included. Different letters in the columns (a–e) indicate significant differences (*p* < 0.05).

**Table 2:** Global antioxidant response (GAR) for Ca(II)-alginate-based beads, with and without cowpea pod extract and for non-encapsulated extract.

	Global Antioxidant Response (GAR)			
	System	Folin–Ciocalteu (mmol_GAE_/kg_product_)	TEAC_ABTS_ (mmol_TROLOX_/kg_product_)	TEAC_FRAP_ (mmol_TROLOX_/kg_product_)
	E	20 ± 2 ^c^	17.1 ± 0.4 ^b^	9.4 ± 0.1 ^b^
Beads with extract	A	47 ± 1 ^b,A^	51 ± 4 ^a,A^	25 ± 1 ^a,A^
	AAG	50 ± 2 ^a,b,A^	52 ± 3 ^a,A^	24.8 ± 0.5 ^a,A^
	AGG	49 ± 2 ^a,b,A^	48 ± 2 ^a,A^	22.0 ± 0.7 ^a,A^
	AP2:1	52.3 ± 0.5 ^a,b,A^	50.9 ± 0.8 ^a,A^	26.7 ± 0.3 ^a,A^
	AP1:1	54 ± 2 ^a,A^	54 ± 6 ^a,A^	26 ± 1 ^a,A^
Beads without extract	A	45 ± 2 ^b,A^	35 ± 1 ^b,B^	20.1 ± 0.3 ^a,B^
	AAG	43.6 ± 0.7 ^b,B^	28 ± 1 ^c,B^	20.3 ± 0.6 ^a,B^
	AGG	43 ± 2 ^b,B^	32 ± 3 ^b,c,B^	20.5 ± 0.8 ^a,A^
	AP2:1	45 ± 1 ^b,B^	35 ± 2 ^b,B^	20 ± 1 ^a,B^
	AP1:1	49.7 ± 0.5 ^a,A^	51 ± 1 ^a,A^	22.3 ± 0.3 ^a,B^

A: alginate; AG: arabic gum; GG: guar gum; P: isolated cowpea protein in ratios of 2:1 and 1:1 (alginate:protein); E: non-encapsulated extract. Different lowercase superscript letters (a–c) indicate significant differences among beads within each group. Different uppercase letters (A–B) indicate significant differences between beads with the same formulation with and without extract. E was only compared to beads with extract (*p* < 0.05).

## Data Availability

The original contributions presented in the study are included in the article/Supplementary Materials; further inquiries can be directed to the corresponding authors.

## Supplementary Materials

The following supporting information can be downloaded at: https://www.mdpi.com/article/10.3390/foods13193071/s1, Antioxidant Capacity Methods; Compression Assay; Table S1: Beads formulation used for the different Ca(II)–alginate systems. Table S2: Morphology analysis of beads containing cowpea pod extract; Table S3: Transversal (T_21_ and T_22_) relaxation times and their corresponding amplitudes of Ca(II)–alginate beads with or without cowpea extract undigested or in gastric and intestinal phases. Figure S1: Mechanical strength by texture analyzer obtained for Ca(II)–alginate beads systems undigested and after the intestinal phase.
