# Supplementary material for: In Vitro Digestion and Fermentation of Cowpea Pod Extracts and Proteins Loaded in Ca(II)-Alginate Hydrogels
Source: Foods. 2024 Sep 26;13(19):3071. doi: 10.3390/foods13193071 (PMC11475951; doi:10.3390/foods13193071)
Supplement: Supplementary file 1 [file foods-13-03071-s001.zip › foods-3218127-supplementary.pdf]

foods

MDPI

Article – Supplementary Information

In Vitro Digestion and Fermentation of Cowpea Pod Extracts and Proteins Loaded in Ca(II)-Alginate Hydrogels

Maria Victoria Traffano-Schiffo [1], Tatiana Rocio Aguirre-Calvo [2,3], Beatriz Navajas-Porras [4], María Victoria Avanza [1], José Ángel Rufián-Henares [4,5,*] and Patricio Román Santagapita [2,3,*]

[1] Instituto de Química Básica y Aplicada del Nordeste Argentino, IQUIBA-NEA, UNNE-CONICET, Avenida Libertad 5460, Corrientes 3400, Argentina; m.traffanoschiffo@conicet.gov.ar (M.V.T.-S.); maria.victoria.avanza@comunidad.unne.edu.ar (M.V.A.)
[2] Departamento de Química Orgánica y Química Inorgánica, Facultad de Ciencias Exactas y Naturales, Universidad de Buenos Aires, Buenos Aires 1428, Argentina; traguic@gmail.com
[3] Centro de Investigaciones en Hidratos de Carbono (CIHIDECAR), Universidad de Buenos Aires-CONICET, Buenos Aires 1428, Argentina
[4] Departamento de Nutrición y Bromatología, Instituto de Nutrición y Tecnología de Alimentos, Centro de Investigación Biomédica, Universidad de Granada, 18011 Granada, Spain; beatriznavajas@ugr.es
[5] Instituto de Investigación Biosanitaria ibs.GRANADA, Universidad de Granada, 18014 Granada, Spain
* Correspondence: jarufian@ugr.es (J.Á.R.-H.); patricio.santagapita@qo.fcen.uba.ar (P.R.S.)

Materials & Methods

1.1. Antioxidant Capacity Methods

a. Folin-Ciocalteu assay

Total Phenolic content (TPC) was determined following the method proposed by Singleton, Orthofer and Lamuela-Raventós [1]. Briefly, 30 μL of each sample mixed with 190 μL of bidistilled water, 15 μL of Folin-Ciocalteu reagent and 60 μL of Na_2CO_3 10% (w/v) were added in triplicate in a 96-well microplate and measured in a microplate reader (FLUOStar Omega, BMG Labtech, Ortenberg, Germany) and the reaction was monitored at 37 °C for 30 min. The calibration curve was prepared using gallic acid in a range of concentration of 0.1 to 2.5 mg/mL. Results were expressed as $mmol_{GAE}/kg_{product}$.

b. Trolox equivalent antioxidant capacity assays

Antioxidant capacity (AC) against $ABTS^{*+}$ radical ($TEAC_{ABTS}$) assay was performed according to Re et al. [2] method with little modifications. Briefly, the $ABTS^{*+}$ radical was obtained by reacting 7 mM ABTS with 2.45 mM potassium persulfate at 25 °C and in the dark during at least 16 h before its use. To prepare the daily working solution, $ABTS^{*+}$ with a solution of ethanol:bi-distilled water (50:50) to obtain an absorbance of 0.70 (±0.02) at 734 nm. Then, 20 μL of each fraction was added in triplicate to a 96-well microplate and mixed with 280 μL of $ABTS^{*+}$ reagent and the reaction was monitored at 37 °C during 20 min using a microplate reader (FLUOStar Omega, BMG Labtech, Ortenberg, Germany). The calibration curve was performed with Trolox (0.01-0.1 mg/mL), and the results were expressed as $mmol_{TROLOX}/kg_{product}$.

Trolox equivalent antioxidant capacity against referred to reducing capacity ($TEAC_{FRAP}$) assay was obtained according to Benzie and Strain [3]. The ferric reduction capacity was determined when 20 μL of each fraction was added in triplicate to a 96-well microplate reacting with 280 μL of (recently prepared) FRAP reagent. The reaction was followed by a microplate reader (FLUOStar Omega, BMG Labtech, Ortenberg, Germany) at 37 °C during 30 min. In the same way as for $TEAC_{ABTS}$, the calibration curve was carried out with Trolox (0.01-0.1 mg/mL), and the results were expressed as $mmol_{TROLOX}/kg_{product}$.

1.2. Compression assay

The resistance to compression of each Ca(II)-alginate bead formulation was determined with the maximum force applied to each system before and after being subjected to digestion fluids. An Instron Testing Machine model 3345 (Canton, MA, USA) equipped with a 50 N load cell connected to Bluehill 2 v. 2.17 software (Canton, MA, USA) was coupled to a flat ending probe of 3.0 mm of diameter to compress the beads at a fixed crosshead speed of 1 mm/min. A force of up to 50% of compression was registered. Ten beads were analyzed for each measurement.

Table S1. Beads formulation used for the different Ca(II)-alginate systems.

System	**Alginate (1.5%)**	**Arabic Gum (0.25 %)**	**Guar Gum (0.25 %)**	**Isolated cowpea protein (ratio 2:1)***	**Isolated cowpea protein (ratio 1:1)***
A	X				
AAG	X	X			
AGG	X		X		
AP2:1	X			X	
AP1:1	X				X

*alginate:isolated cowpea protein ratio.

Table S2. Morphology analysis of beads containing cowpea pod extract.

System	**Area (cm^2)**	**Circularity**	**Perimeter (cm)**	**Feret's diameter (cm)**
A	0.052 ± 0.005 ^{c}	0.79 ± 0.08 ^{c}	0.92 ± 0.09 ^{c}	0.29 ± 0.02 ^{b}
AAG	0.056 ± 0.005 ^{b}	0.73 ± 0.03 ^{d}	0.98 ± 0.06 ^{b}	0.29 ± 0.02 ^{b}
AGG	0.062 ± 0.006 ^{a}	0.66 ± 0.06 ^{e}	1.09 ± 0.08 ^{a}	0.31 ± 0.02 ^{a}
AP2:1	0.050 ± 0.003 ^{d}	0.84 ± 0.05 ^{a}	0.87 ± 0.04 ^{d}	0.27 ± 0.01 ^{c}
AP1:1	0.057 ± 0.004 ^{b}	0.82 ± 0.04 ^{b}	0.94 ± 0.04 ^{c}	0.29 ± 0.01 ^{b}

*A, alginate; AG, arabic gum; GG, guar gum and P, cowpea protein. Standard deviation values are included. Different letters on the columns (a–e) indicate significant differences ($p < 0.05$).

Table S3. Transversal (T_{21} and T_{22}) relaxation times and their corresponding amplitudes of Ca(II)-alginate beads with or without cowpea extract undigested or in gastric and intestinal phases. A: alginate; AG: arabic gum; GG: guar gum; P: isolated cowpea protein in relations 2:1 and 1:1 (alginate:protein).

	Undigested beads		Gastric Phase		Intestinal Phase	
	A_{21} (%)	T_{21} (ms)	A_{21} (%)	T_{21} (ms)	A_{21} (%)	T_{21} (ms)
**A**	75.0 ± 2 ^{d,B}	90.0 ± 0.2 ^{c,A}	89.3 ± 0.2 ^{c,A}	49.5 ± 0.5 ^{b,B}	2.5 ± 0.3 ^{a,b,C}	47.0 ± 3 ^{a,B}
**AAG**	80.0 ± 0.7 ^{c,B}	93.0 ± 1 ^{b,A}	89.7 ± 0.03 ^{b,c,A}	55.4 ± 0.3 ^{a,B}	1.1 ± 0.2 ^{c,C}	24.0 ± 5 ^{b,C}
**AGG**	80.0 ± 1 ^{b,c,B}	97.8 ± 0.08 ^{a,A}	89.6 ± 1 ^{c,A}	54.3 ± 0.6 ^{a,B}	3.0 ± 1 ^{a,C}	44.0 ± 6 ^{a,C}
**AP2:1**	83.9 ± 0.7 ^{b,B}	72.9 ± 0.9 ^{d,A}	92.0 ± 0.8 ^{a,b,A}	40.8 ± 0.3 ^{c,B}	1.7 ± 0.05 ^{b,C}	16.1 ± 0.2 ^{b,C}
**AP1:1**	87.0 ± 0.3 ^{a,B}	60.9 ± 0.5 ^{e,A}	93.0 ± 0.5 ^{a,A}	36.2 ± 0.9 ^{d,B}	1.9 ± 0.2 ^{b,C}	10.2 ± 0.3 ^{c,C}
**EA**	77.0 ± 2 ^{c,B}	73.0 ± 1 ^{a,A}	92.0 ± 0.01 ^{a,A}	48.7 ± 0.5 ^{b,B}	2.2 ± 0.4 ^{a,b,C}	29.0 ± 7 ^{a,b,C}
**EAAG**	82.0 ± 2 ^{b,B}	76.2 ± 0.9 ^{a,A}	91.0 ± 1 ^{a,b,A}	49.7 ± 0.5 ^{a,b,B}	1.6 ± 0.5 ^{b,C}	20.9 ± 0.7 ^{a,C}
**EAGG**	84.0 ± 1 ^{a,b,B}	73.9 ± 0.9 ^{ab,A}	90.8 ± 0.08 ^{b,A}	52.0 ± 1 ^{a,B}	1.3 ± 0.2 ^{b,C}	27.0 ± 2 ^{a,C}
**EAP2:1**	90.7 ± 0.4 ^{a,A}	58.9 ± 0.3 ^{c,A}	92.8 ± 0.4 ^{a,A}	43.6 ± 0.7 ^{c,B}	1.8 ± 0.06 ^{b,B}	14.5 ± 0.5 ^{c,C}
**EAP1:1**	84.7 ± 0.3 ^{b,B}	62.4 ± 0.3 ^{b,A}	92.1 ± 0.8 ^{a,b,A}	41.0 ± 1 ^{c,B}	3.6 ± 0.5 ^{a,C}	17.2 ± 0.8 ^{b,c,C}

	Undigested beads		Gastric Phase		Intestinal Phase	
	A_{22} (%)	T_{22} (ms)	A_{22} (%)	T_{22} (ms)	A_{22} (%)	T_{22} (ms)
**A**	25.0 ± 2 ^{a,B}	235 ± 13 ^{b,A}	10.7 ± 0.2 ^{a,C}	234 ± 46 ^{a,A}	97.5 ± 0.3 ^{a,A}	163 ± 6 ^{a,B}
**AAG**	19.9 ± 0.7 ^{b,B}	307 ± 17 ^{a,A}	10.3 ± 0.03 ^{a,C}	288 ± 20 ^{a,A}	98.9 ± 0.2 ^{a,A}	122 ± 22 ^{a,b,B}
**AGG**	20.0 ± 1 ^{b,B}	312 ± 31 ^{a,A}	10.0 ± 1 ^{a,C}	251 ± 16 ^{a,A}	97.0 ± 1 ^{a,A}	141 ± 7 ^{a,B}
**AP2:1**	16.2 ± 0.7 ^{b,B}	334 ± 38 ^{a,A}	8.0 ± 0.8 ^{b,C}	198 ± 16 ^{a,B}	98.3 ± 0.05 ^{a,A}	82.0 ± 12 ^{b,C}

**AP1:1**	12.8 ± 0.3 ^{c,B}	297 ± 0.8 ^{a,A}	7.0 ± 0.5 ^{c,C}	186 ± 49 ^{a,B}	98.1 ± 0.2 ^{a,A}	75.7 ± 0.2 ^{b,C}
**EA**	23.0 ± 2 ^{a,B}	155 ± 7 ^{c,A}	8.0 ± 0.01 ^{b,c,C}	190 ± 52 ^{a,b,A}	97.8 ± 0.4 ^{a,b,c,A}	109 ± 0.3 ^{b,A}
**EAAG**	18.0 ± 2 ^{b,B}	174 ± 9 ^{c,A}	9.0 ± 1 ^{a,b,c,C}	175 ± 62 ^{a,b,A}	98.4 ± 0.5 ^{a,b,A}	104 ± 14 ^{a,b,c,A}
**EAGG**	16.0 ± 1 ^{b,B}	171 ± 10 ^{c,B,C}	9.2 ± 0.08 ^{a,C}	223 ± 66 ^{a,A}	98.7 ± 0.2 ^{a,b,A}	128 ± 1 ^{a,C}
**EAP2:1**	9.3 ± 0.4 ^{c,B}	223 ± 12 ^{b,A}	7.2 ± 0.4 ^{c,B}	168 ± 65 ^{b,A,B}	98.2 ± 0.06 ^{b,A}	123 ± 48 ^{a,b,c,B}
**EAP1:1**	15.3 ± 0.2 ^{b,B}	356 ± 15 ^{a,A}	7.9 ± 0.8 ^{a,b,c,C}	171 ± 35 ^{a,b,B}	96.4 ± 0.5 ^{c,A}	72.6 ± 0.3 ^{c,C}

Different lowercase letters (a-d) indicate significant differences between each group of Ca(II)-alginate systems (controls and with extracts); different capital letters (A-C) indicate significant differences comparing the digestion phases for the same bead system ($p < 0.05$).

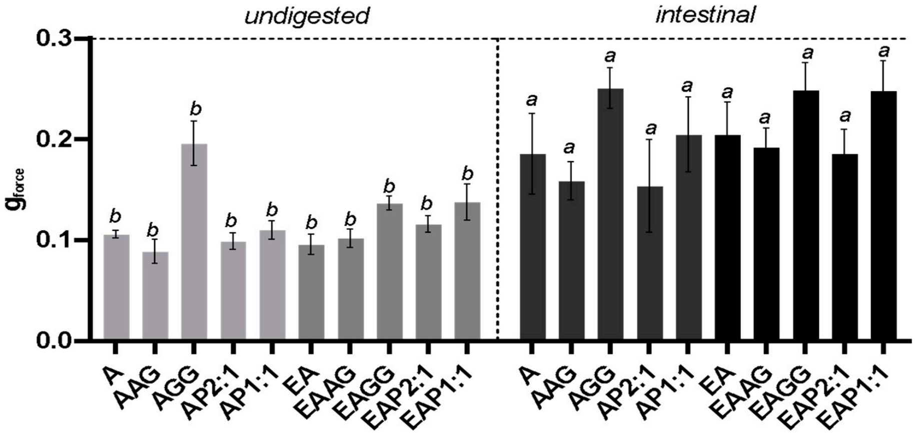

Figure S1. Mechanical strength by texture analyzer obtained for Ca(II)-alginate beads systems undigested and after the intestinal phase. Mean and standard deviation are included. Different lowercase letters (a-b) indicate significant differences between Ca(II)-alginate systems undigested and after digestion ($p < 0.05$).

1. Singleton, V.L.; Orthofer, R.; Lamuela-Raventós, R.M. Analysis of total phenols and other oxidation substrates and antioxidants by means of folin-ciocalteu reagent. *Method. Enzymol.* **1999**, *299*, 152–178. https://doi.org/10.1016/S0076-687999017-1.
2. Re, R.; Pellegrini, N.; Proteggente, A.; Pannala, A.; Yang, M.; Rice-Evans, C. Antioxidant activity applying an improved ABTS radical cation decolorization assay. *Free Radic. Biol. Med*. **1999**, *26*, 1231−1237. https://doi.org/10.1016/S0891-5849(98)00315-3.
3. Benzie, I.F.; Strain, J.J. The ferric reducing ability of plasma (FRAP) as a measure of "antioxidant power": The FRAP assay. *Anal. Biochem.* **1996**, *239*, 70–76. https://doi.org/10.1006/abio.1996.0292.
